# The Effect of Different Coffee Cherry Pulp-Retention Amounts on the Quality of Robusta Green Coffee Beans

**DOI:** 10.3390/foods15152714

**Published:** 2026-08-01

**Authors:** Rongjie Gui, Xinyu Tang, Kejing Zhao, Han Yang, Zixin Chen, Yulan Li, Yijiaqi Zhang, Jiayi Shi, Ang Zhang, Xuejun Li, Yanli Huang

**Affiliations:** 1 College of Tropical Crops, Yunnan Agricultural University, Pu’er 665000, China; 18387915827@163.com (R.G.); 13187778876@163.com (X.T.);; 2 Yunnan Key Laboratory of Coffee (YAU), Pu’er 665000, China; 3 Spice and Beverage Research Institute, Chinese Academy of Tropical Agricultural Sciences, Wanning 571533, China; 4 International Joint Research Center for Tropical Spice and Beverage, Wanning 571533, China; 5 Coffee Hainan Engineering Research Center (In Preparation), Wanning 571533, China; 6 Pu’er Coffee Innovation Center, Pu’er 665000, China; 7 Hainan Provincial Key Laboratory of Genetic Improvement and Quality Regulation for Tropical Spice and Beverage Crops, Wanning 571533, China

**Keywords:** Robusta coffee, coffee cherry pulp retention, honey processing, volatile flavor precursors, grey relational analysis

## Abstract

Robusta coffee (*Coffea canephora*) possesses great industrial potential, whereas its intrinsic flavor defects compromise market competitiveness. Honey processing utilizing residual pulp and mucilage is capable of improving green coffee quality; nevertheless, the absence of quantitative research regarding graded pulp retention limits its industrial application. In this study, fermentation experiments with gradient retention of coffee cherry pulp were conducted to clarify the differential regulatory effects of varying coffee cherry pulp-retention amounts on physicochemical properties, functional components and volatile flavor precursor compounds of Hainan Robusta coffee. Quantitative scoring of coffee quality using international standard cup measuring instruments and the grey relational analysis was further employed to comprehensive quality assessment. Compared with the 0% coffee cherry pulp-retention treatment, all other treatments showed higher levels of selected functional components and volatile flavor precursor compounds. The 66% coffee cherry pulp-retention treatment enriched key roasting flavor substrates: sucrose, organic acids, pyrazines, and esters were increased by 8.74%, 26.58%, 80.66% and 30.18%, respectively. Meanwhile this treatment exhibited significantly lower protein and alkaloid contents than the 100% and 33% coffee cherry pulp-retention treatments, and significantly lower flavonoid content than the 0% and 33% coffee cherry pulp-retention treatments. This treatment exhibited remarkably excellent cup scores by 80.35 and superior comprehensive quality, outperforming the other treatments by 6.05–18.51%. This study identifies the optimal coffee cherry pulp-retention ratio for Robusta coffee post-harvest honey processing, providing theoretical basis and feasible technical references for the quality improvement, standardized processing and industrial promotion of local Robusta coffee.

## 1. Introduction

Robusta coffee (*Coffea canephora*) is one of the core categories in the global coffee industry, accounting for approximately 40% to 43% of total global coffee production. It serves as a pillar cash crop for low-altitude tropical countries such as Vietnam, Brazil, Indonesia, and Uganda. It plays a crucial strategic role in ensuring the stability of the global coffee supply chain and maintaining the industry’s supply-demand balance [1,2]. The Robusta variety offers significant cultivation advantages, including heat tolerance, high resistance to pests and diseases, and high yields per unit area, resulting in stable economic returns [3]. However, it also has inherent quality flaws, a strong bitterness and a lack of aroma which severely limit its applications. Therefore, it has long been confined to use in instant coffee, dark-roast blends, and industrial extraction, and its economic value is far lower than that of Arabica coffee [4,5]. The current global coffee consumption growth rate remains at 3.5% per annum, and market demand for diverse coffee flavors and health benefits is driving the transformation of Robusta coffee toward specialty and differentiated products [6,7]. The quality improvement and industrial transformation of Robusta coffee primarily rely on two approaches: fundamental-level improvement through germplasm innovation and optimization of cultivation techniques, and post-harvest and primary processing technology improvements to enhance quality [8]. In particular, optimizing primary processing techniques can precisely address the sensory defects of Robusta coffee and unlock its potential functional value [9]. This makes it a key strategy for improving the quality of Robusta coffee and enhancing the commercial value of the product.

A ripe coffee cherry is a multilayered drupe composed of exocarp, mesocarp, endocarp, seed coat and seed kernel. Among all tissue layers, only the seed kernel constitutes green coffee beans, the primary raw material underlying coffee processing and flavor formation, whose chemical profile exerts a decisive influence on the finished coffee quality [10]. Green coffee beans are rich in nutrients such as proteins, carbohydrates, and fats, as well as key bioactive components including alkaloids, organic acids, polyphenols, and flavonoids. They are also rich in volatile flavor precursor compounds such as alcohols, phenols, pyrroles, pyrazines, esters, and indoles, which constitute their core chemical composition. Among these, polyphenols possess strong antioxidant activity and can modulate the aromatic complexity and bitter texture profile of coffee after roasting; sugars and organic acids are the primary sources of coffee’s sweet and sour flavors, helping to balance the beverage’s mouthfeel and enhance its flavor richness. From the perspective of the quality evaluation system, the quality of green coffee beans is primarily determined by a combination of flavor precursor components and sensory quality compounds [7,11,12]. Volatile flavor precursor compounds, including lipids, saccharides, chlorogenic acids and caffeine, act as substrates for the Maillard reaction and caramelization during roasting, which lays the fundamental aromatic and flavor framework of coffee. Sensory-active components such as organic acids and volatile aromatics directly modulate coffee’s acidity, sweetness, cleanliness, body and aroma complexity, serving as core criteria to evaluate the commercial value and quality grade of green coffee beans [13].

Currently, the primary processing methods for Robusta coffee include three traditional techniques: washed, natural, and honey process [14]. These different processing methods result in significant differences in the retention or removal of the mucilage and pulp layers, which in turn directly alter the metabolic environment and flavor profile of the green coffee beans [14,15]. Coffee cherry pulp is abundant in bioactive components including free amino acids, carbohydrates, soluble sugars, organic acids and polyphenols, and hosts abundant indigenous microbial communities. Endogenous components within the coffee cherry pulp supply critical substrates for post-harvest metabolism and fermentation reactions. Epiphytic microorganisms have been reported to participate in post-harvest metabolism and fermentation and may contribute to coffee quality variation [16,17,18]. It is evident that the state of coffee cherry pulp is a key variable that determines the sensory quality and chemical characteristics of green coffee beans, and it has a significant impact on improving coffee quality and efficiency. Among various primary processing techniques, the controlled mixed fermentation process via honey processing with coffee cherry pulp retention not only promotes the accumulation of flavor precursors through enzymatic reactions but also simultaneously reduces bitter taste perception through the isomerization of chlorogenic acids [19,20,21]. Consequently, it has become a new technological approach for improving coffee quality defects and overcoming the limitations of traditional processing methods. Coffee cherry pulp contains carbohydrates, organic acids, and other bioactive constituents that may alter the physicochemical environment during coffee processing. Previous studies have suggested that pulp retention may be associated with changes in flavor precursor composition and perceived bitterness [15,22,23]. Compared with conventional washed processing, retention of coffee cherry pulp and mucilage has been reported to be associated with differences in fermentation conditions and volatile flavor-related compounds, including esters [24].

However, current research on quality control in coffee cherry fermentation and primary processing has primarily focused on Arabica coffee, with study designs largely limited to comparisons between full coffee cherry pulp sun-drying and hulled washed processing. There is a lack of gradient-based, quantitative investigations into the underlying processing mechanisms [25]. Particularly for Robusta coffee, the dose-dependent effects of different coffee cherry pulp-retention ratios within controlled fermentation systems and the associated quality regulation mechanisms remain largely unclear. Moreover, existing studies have predominantly been conducted at the laboratory scale, and the process stability, reproducibility, and applicability for industrial-scale standardization have yet to be adequately validated. Therefore, the present study was conducted using Robusta coffee (*Coffea canephora*) sourced from Hainan Island, China, as the experimental material. A multi-gradient coffee cherry pulp supplementation approach was designed to carry out mixed aerobic fermentation experiments on green coffee beans; systematically determine the moisture content, hardness, crude protein, sucrose, total soluble sugars, crude fat, and other physicochemical quality indicators; analyze the content of functional components such as total alkaloids, total organic acids, total flavonoids, and total polyphenols; simultaneously detect key bioactive compounds such as fenugreek alkaloids, caffeine, theobromine, and cryptochlorogenic acid; and comprehensively characterize the spectrum of volatile flavor precursor compounds in the samples. Furthermore, the present study employed the grey relational analysis method to integrate multidimensional quality indicators and construct a comprehensive quality evaluation system for green coffee beans [26]. The findings will elucidate the regulatory effects of different coffee cherry pulp-retention treatments on the quality of Robusta green coffee beans, and to reveal the underlying mechanisms by which coffee cherry pulp-mediated fermentation improves bean quality. This study aims to provide theoretical support and technical references for quality improvement of Robusta coffee, optimization of primary processing techniques, and the establishment of standardized production systems.

## 2. Materials and Methods

### 2.1. Sample Preparation

The experimental materials used in this study were mature fresh cherries of Robusta coffee (*Coffea canephora*), uniformly harvested in January 2026 from healthy plants of the same batch at Xinglong Tropical Botanical Garden, Wanning City, Hainan Province, China. The selection criteria were strictly defined as fully ripe cherries with bright red to dark red coffee cherry pulp, uniform development, and free from mechanical damage, mold contamination, or insect infestation. Immediately after harvest, unripe, overripe, and floating defective fruits, as well as any foreign matter, were removed, and the remaining fruits were homogenized to ensure consistency between batches during subsequent processing and fermentation experiments.

### 2.2. Experimental Design

This experiment employed a one-factor experimental design, with coffee cherry pulp-retention rate as the sole variable. A total of four treatments were established, each with three independent biological replicates. Four treatments were established: high pulp retention (H), medium pulp retention (M), low pulp retention (L), and a no-pulp control (N). The fresh-weight ratios of coffee cherry pulp retention were 1.5:1, 1.0:1, 0.5:1, and 0:1 for the H, M, L, and N treatments, respectively. The H treatment represented the initial full-pulp level (100%) at the beginning of processing. Accordingly, the M and L treatments retained approximately 66% and 33%, respectively, of the initial full coffee cherry pulp amount, whereas the N treatment contained no retained coffee cherry pulp (0%).

All treatments were processed simultaneously at the same experimental site and followed an identical daily processing schedule to minimize variation in environmental exposure and handling. During the daytime, the materials were spread into a thin and even layer for sun-drying. All treatment piles were maintained under the same outdoor environmental conditions to ensure comparable temperature and relative-humidity conditions during fermentation. At night, the materials were gathered into outdoor piles to allow aerobic fermentation. The pile core temperature increased progressively during nighttime piling and was maintained below 50 °C; in practice, the core temperature was generally maintained within 40–50 °C. When the pile temperature approached or exceeded the upper limit, the piles were manually turned, flattened, or reduced in height to promote heat dissipation and maintain comparable fermentation conditions among treatments. The materials were spread again on the following day, resulting in a repeated day-drying/night-piling cycle [27].

Upon completion of fermentation, all treatments were terminated after 25 days of uniform fermentation. During the fermentation period, the material was spread out to dry during the day and composted in piles at night; the height of the compost piles was adjusted periodically to ensure that the fermentation temperatures remained similar across all treatments. All experimental samples were processed following identical procedures and finally placed on raised drying beds for uniform drying until the moisture content of the green beans stabilized at approximately 12% [2], at which point sample preparation was completed.

### 2.3. Physicochemical Quality of Green Coffee Beans

#### 2.3.1. Moisture Content Determination

Moisture content of the samples was determined using the constant-temperature drying method. An accurately weighed 1.00 g portion of the sample was placed in a rapid moisture analyzer (HB43-S, Mettler Toledo, Greifensee, Switzerland) and dried at 115 °C for 40 min until constant weight was achieved. The moisture content was then calculated based on the weight loss [28].

#### 2.3.2. Hardness Measurement of Green Coffee Beans

The hardness of green coffee beans was measured using a texture analyzer (TA.XTC-18, Baosheng Technology, Shanghai, China) equipped with a P/5 probe (2 mm in diameter). The minimum indentation force was set to 20 N, the penetration depth to 10.0 mm, and the test speed to 1.0 mm/s. All samples were tested under these uniform parameters.

#### 2.3.3. Crude Protein Content Analysis of Green Coffee Beans

Crude protein content was determined using the Kjeldahl method. The protein content P (%) was calculated according to the following formula:

(1) $$P\;=\;\left(A-B\right)\;\times \;C\;\times \;14\;\times \;\frac{V_{1}}{\left(M\;\times \;V_{2}\right)}\;\times \;6.25$$

where A is the volume of hydrochloric acid consumed during the sample titration, B is the volume of hydrochloric acid consumed during the blank titration, C is the standard concentration of hydrochloric acid, 14 is the nitrogen equivalent mass, V_1_ is the total volume of digestion solution, M is the sample mass, V_2_ is the volume of digestion solution used for distillation, and 6.25 is the nitrogen-to-protein conversion factor.

#### 2.3.4. Sugar Content Analysis of Green Coffee Beans

Total sugar and sucrose content were determined using near-infrared spectroscopy (NIRS) with a Foss 6500 spectrometer (Foss NIRSystems, Laurel, MD, USA) equipped with a diffuse reflectance detector. The spectral scan range was 400–2500 nm, and spectral reflectance was recorded in log(1/R) mode. The data were processed using WinISI 4.5 software (Infrasoft International, Port Matilda, PA, USA) according to the method described by Barbosa et al. [9].

#### 2.3.5. Crude Fat Content Analysis of Green Coffee Beans

Crude fat content was determined using the Soxhlet extraction method [29]. The crude fat content T was calculated according to the following formula.

(2) $$T=\frac{\left(W_{2}-W_{3}\right)}{\left(W_{2}-W_{1}\right)}\times 100$$

where W_1_ is the mass of the defatted filter paper, W_2_ is the total mass of the defatted filter paper and the sample, and W_3_ is the total mass of the sample and the defatted filter paper after defatting.

### 2.4. Functional Component Analysis

#### 2.4.1. Alkaloids and Organic Acids Profiling

Ground coffee powder (5.0 g) was extracted with 60 mL of ultrapure water under reflux at 70 °C for 2.5 h. After centrifugation at 4000 rpm for 10 min, the supernatant was adjusted to 100 mL and filtered through a 0.22 μm membrane. An aliquot (400 μL) was vacuum-dried and reconstituted in 300 μL of methanol–acetonitrile–water (2:2:1, *v*/*v*/*v*) containing 5 μg/mL 2-chlorophenylalanine. After vortexing for 30 s, homogenization at 55 Hz for 60 s, sonication for 10 min, and incubation at −20 °C for 30 min, the sample was centrifuged at 12,000 rpm and 4 °C for 10 min and filtered through a 0.22 μm membrane [30].

Chromatographic separation was performed using a Vanquish Flex UHPLC system (Thermo Fisher Scientific, Waltham, MA, USA) equipped with an ACQUITY UPLC HSS T3 column (Waters Corporation, Milford, MA, USA) (2.1 × 100 mm, 1.8 μm) at 40 °C. The mobile phases were water containing 0.1% formic acid (A) and acetonitrile containing 0.1% formic acid (B), at a flow rate of 0.4 mL/min. The gradient was as follows: 0–0.5 min, 2% B; 0.5–4.7 min, 2–95% B; 4.7–6.0 min, 95% B; 6.0–6.1 min, 95–2% B; and 6.1–8.0 min, 2% B.

Mass spectra were acquired using a mass spectrometer equipped with an electrospray ionization (ESI) source operating in positive- and negative-ion modes (ESI+/ESI−). The four most intense precursor ions were selected for HCD fragmentation at 30% normalized collision energy, with an MS/MS resolution of 15,000 and a dynamic exclusion time of 4 s. Metabolites were annotated by matching retention time, accurate mass (mass error < 10 ppm), MS/MS fragmentation patterns, and collision energy against an in-house spectral library and public databases, including HMDB, METLIN, MassBank, and mzCloud. All annotations were manually reviewed, and only metabolites identified at MSI confidence Level 2 or higher were retained. Relative abundances were expressed as internal-standard-normalized peak areas.

#### 2.4.2. Total Polyphenols Profiling

Total polyphenols were determined by the Folin–Ciocalteu colorimetric method [31,32]. A 0.20 g sample of ground coffee powder was extracted at room temperature with 5 mL of 70% (*v*/*v*) methanol using ultrasonication for 15 min, followed by centrifugation at 5000 rpm for 10 min. The extraction was repeated twice, and the combined extracts were collected. Then, 1.0 mL of coffee extract was reacted with 5.0 mL of 10% (*v*/*v*) Folin–Ciocalteu reagent for 5 min, followed by the addition of 4.0 mL of 7.5% Na_2_CO_3_ solution. After reacting at room temperature for 60 min, the absorbance was measured at 765 nm. The total polyphenol content is expressed as gallic acid equivalents. The standard curve is y = 0.0069x − 0.0003 (R^2^ = 0.9955). Results were expressed as mg gallic acid equivalents per gram of sample (mg GAE/g).

#### 2.4.3. Total Flavonoids Profiling

Total flavonoids were determined by a colorimetric method [33]. A 0.50 g sample of ground coffee was extracted with 25 mL of 60% ethanol (*v*/*v*) under ultrasonic irradiation at 40 °C for 15 min, followed by centrifugation at 5000× *g* for 15 min. The extraction was repeated twice, and the combined extracts were collected. Total flavonoid content was determined using a colorimetric method. Briefly, 2.0 mL of coffee extract was sequentially mixed with 3 mL of 60% ethanol and 1 mL of NaNO_2_ solution (50 mg/mL). After 6 min of reaction, 1.5 mL of Al(NO_3_)_3_ solution was added and the mixture was allowed to react for 15 min. The absorbance was measured at 510 nm. Total flavonoid content was expressed as rutin equivalents, and the calibration curve was y = 3.4369x − 0.0023 (R^2^ = 0.9985). Results were expressed as mg rutin equivalents per gram of sample (mg RE/g).

### 2.5. Bacterial Community DNA Extraction, Sequencing, and Bioinformatics Analysis

At the end of the 25-day fermentation–drying process, surface-associated fermentation materials were aseptically collected from a standardized amount of coffee beans from each biological replicate of the H, M, L, and N treatments (*n* = 3 per treatment). For the H, M, and L treatments, the collected materials included pulp and mucilage residues adhering to the coffee beans, whereas bean-surface materials from the washed coffee beans were collected from the N treatment using the same procedure. Total DNA was extracted from 0.5 g of each homogenized sample using the E.Z.N.A.^®^ Soil DNA Kit (Omega Bio-tek, Norcross, GA, USA) according to the manufacturer’s instructions. DNA concentration and purity were assessed using a NanoDrop ND-2000 spectrophotometer (Thermo Fisher Scientific, Waltham, MA, USA), and DNA integrity was verified by 1.0% agarose gel electrophoresis.

The V3–V4 region of the bacterial 16S rRNA gene was amplified using the primers 338F (5′-ACTCCTACGGGAGGCAGCAG-3′) and 806R (5′-GGACTACHVGGGTWTCTAAT-3′). PCR was performed in a 25 μL reaction containing 12.5 μL of 2× Phusion High-Fidelity PCR Master Mix, 0.2 μM of each primer, and approximately 10 ng of template DNA. The amplification program consisted of initial denaturation at 98 °C for 30 s, followed by 25 cycles of 98 °C for 10 s, 50 °C for 30 s, and 72 °C for 30 s, with a final extension at 72 °C for 5 min. Triplicate PCR products from each sample were pooled, purified, quantified, and mixed in equimolar proportions. Paired-end sequencing was performed on an Illumina MiSeq PE300 platform (Illumina, Inc., San Diego, CA, USA) [34,35].

Raw paired-end reads were processed using QIIME 2 version 2023.2 with the DADA2 plugin [36]. Primer sequences and low-quality bases were removed using trimming and truncation parameters selected according to the per-base sequence-quality profiles. DADA2 was used for sequence denoising, paired-end merging, chimera removal, and amplicon sequence variant inference [37]. Taxonomic assignment was performed using a Naive Bayes classifier trained on the 338F/806R-amplified V3–V4 region of the SILVA 138 database, with a confidence threshold of 0.80. Non-bacterial sequences, including chloroplast and mitochondrial sequences, were removed before downstream analysis. The feature table was rarefied to 35,000 reads per sample [38]. Genus-level relative abundances were calculated, and the 15 bacterial genera with the highest mean relative abundances across all samples were used to construct the chord diagram. Alpha diversity of bacterial communities under different coffee cherry pulp-retention treatments was evaluated by calculating the Shannon–Wiener index, Simpson diversity index, dominance index, and evenness index based on the genera accounting for the top 1% of total relative abundance.

### 2.6. Volatile Flavor Precursor Compounds Analysis

Volatile flavor precursor compounds were determined by gas chromatography–mass spectrometry (Agilent-7890B/5977B GC–MS, Agilent Technologies Inc., Palo Alto, CA, USA), which included a headspace solid phase microextraction component. A precisely weighed 1.0 g green coffee beans sample and 0.3 g NaCl were mixed and placed in a 20 mL screw vials sealed. A DB-WAX flexible quartz capillary column (Agilent J&W Scientific, Folsom, CA, USA)(30 m × 0.25 mm × 0.25 μm) was used for chromatographic separation. The inlet temperature was set to 250 °C, with an initial temperature of 40 °C for 2 min. The temperature was then increased to 100 °C at 13 °C/min for 2 min, followed by a further increase to 200 °C at 3 °C/min for 1 min, then followed by a further increase to 230 °C at 8 °C/min for 2 min. The carrier gas used was high purity helium (99.999%), with a flow rate of 1 mL/min, and the injection was performed without split [25].

The ion source was operated in EI mode with an ionization energy of 70 eV. The ion source temperature was set to 250 °C. The scanning mass range was 30–350 *m*/*z*, and the scanning speed was 781 u/s. Qualitative analysis was conducted by comparing and searching the National Institute of Standards and Technology (NIST) library L7 spectral library. Semi-quantitative analysis was performed using the internal standard method, with 3-octanol as the internal standard. Content calculation formula:

(3) $$X_{i}\;=\;\frac{V_{s}\;\times \;C_{s}}{M}\;\times \;\frac{I_{i}}{I_{s}}\;\times {\;10}^{-3}$$

In the formula, X_i_ represented the unknown compound content; V_s_ was the volume of the internal standard; C_s_ was the concentration of the internal standard; M was the sample mass; I_s_ was the peak area of the internal standard; and I_i_ was the peak area of an unknown compound.

### 2.7. Grey Relation Analysis

Grey relational analysis (GRA) was used to comprehensively evaluate the quality characteristics of green coffee beans among the different coffee cherry pulp-retention treatments. The evaluation system included selected physicochemical and volatile-compound indicators. The indicators were selected based on four principles: representativeness (covering physicochemical, functional, volatile, and sensory quality dimensions), measurability (analytically determined using established methods), independence (minimizing redundancy among correlated variables), and discriminatory ability (retaining indicators with sufficient variability across treatments). Volatile compounds were selected according to the following criteria: relatively high abundance within their respective chemical classes; recognized importance to coffee aroma quality based on previous reports; and representativeness of major volatile classes. Thus, the selected compounds were intended to capture both the overall volatile profile and aroma-active characteristics while avoiding excessive weighting of low-abundance or infrequently detected compounds. Indicator weights were determined using the entropy weight method, which objectively assigns higher weights to indicators with greater variability (lower entropy) and lower weights to those with limited variation, thereby minimizing subjective bias and ensuring data-driven, reproducible results. Before GRA, all indicator values were normalized to the range of 0–1. For beneficial indicators, for which a higher value indicated better quality, normalization was performed as follows:

(4) $$\varepsilon_{i}\left(k\right)=\frac{\min_{i}\min_{k}\Delta_{i}\left(k\right)\;+\;\rho \max_{i}\max_{k}\Delta_{i}\left(k\right)}{\Delta_{i}\left(k\right)\;+\;\rho \max_{i}\max_{k}\Delta_{i}\left(k\right)}$$

In the formula, $\Delta i\left(k\right)\;=\;\left|X_{0}\left(x\right)-X_{i}\left(k\right)\right|$ represent the absolute difference between $X_{0}$ and $X_{i}$ at k; $\min_{i}\min_{k}\Delta_{i}\left(k\right)$ was the minimum difference at the secondary level, $\rho \max_{i}\max_{k}\Delta_{i}\left(k\right)$ was the maximum difference at the secondary level; ρ was the discrimination coefficient, ρ with smaller values indicating greater differentiation among relational coefficients, thereby enhancing discriminative capacity, typically set to 0.5 within the [0, 1] range. Construct the grey relational degree based on the above formula:

(5) $$\gamma_{i}\;=\;\frac{1}{n}\sum_{k=1}^{n}\varepsilon_{i}\left(k\right)$$

(6) $$\gamma_{i}^{\prime }=\sum_{k=1}^{n}\varepsilon_{i}\left(k\right)\omega_{i}\left(k\right)$$

In the formula, $\gamma_{i}$ was the weighted correlation degree. $\gamma_{i}^{\prime }$ was the equal−weight relational degree, calculate the average value of $\varepsilon_{i}$ to obtain the equal−weighted correlation degree $\gamma_{i}$ of volatile components is obtained. $\gamma_{i}\left(k\right)$ was the weight coefficient. The weights among different indicators were calculated using the entropy weight method, which is employed to minimize the evaluation indicators.

(7) $$H_{j}=-\frac{1}{\mathrm{lnk}}\sum_{i=1}^{k}f_{\mathrm{ij}}\ln f_{\mathrm{ij}}$$

(8) $$\omega_{j}=\frac{1-H_{j}}{k-\sum_{i=1}^{k}H_{j}}$$

In the formula, $H_{j}$ was the entropy value of each evaluation indicator, $\omega_{j}$ was the weight coefficient.

### 2.8. Sensory Evaluation

Sensory evaluation was conducted in a sensory laboratory under controlled conditions in accordance with ISO 8589:2007 [39] guidelines. A trained panel consisting of 20 assessors (16 females and 4 males) was recruited from the Spice and Beverage Research Institute, Chinese Academy of Tropical Agricultural Sciences, Wanning Xinglong Coffee Industry Association, and several coffee processing and retail enterprises located in Wanning City, Hainan Province to evaluate the sensory characteristics of coffee samples prepared under four treatment levels (H, M, L, N). Prior to the formal evaluation, panelists received sensory training focused on coffee attribute recognition, descriptor alignment, score calibration, and evaluation procedures. Ten sensory descriptors were established for sample evaluation: dry aroma, wet aroma, flavor, aftertaste, acidity, sweetness, mouthfeel, bitterness, body, and cleanliness. Each attribute was evaluated using a 10-point scale, where higher scores indicated better sensory quality. Coffee samples were randomly coded with three-digit numbers and served in identical odor-free cups. The presentation order was randomized among panelists to minimize order effects. Panelists were instructed to rinse their palates with water between samples to avoid carryover effects. Three independent sensory evaluation sessions were conducted, and the mean scores were used for subsequent analysis. The total sensory score was calculated as the sum of the scores for the ten sensory attributes, and samples were ranked according to their total scores. Twenty reviewers were invited; to reduce scoring fluctuations caused by personal preferences, two scores with significant outliers were removed.

### 2.9. Data Analysis

All data were first tested for normality using the Shapiro–Wilk test and for homogeneity of variances using Levene’s test prior to one-way analysis of variance (ANOVA). After ANOVA, post hoc comparisons among treatment means were performed using Fisher’s least significant difference (LSD) test at a significance level of *p* < 0.05, and treatment differences were denoted by lowercase letters. Effect sizes were estimated as partial eta-squared (η^2^) to indicate the magnitude of treatment effects. Principal coordinate analysis (PCoA) based on Bray–Curtis dissimilarity was conducted using CANOCO 4.5 (Microcomputer Power, Ithaca, NY, USA) to visualize the compositional similarity of volatile compounds across treatments. Pearson correlation analysis was used to examine the associations among volatile compounds. Genus-level bacterial relative-abundance data were visualized using a chord diagram in R, with ribbon width indicating the contribution of each genus to the corresponding treatment. All statistical analyses, including ANOVA and molecular network analysis, were performed using R version 4.6.0. Figures were generated using Origin 2022 ( OriginLab Corporation, Northampton, MA, USA) and Cytoscape 3.8.2 (Cytoscape Consortium, Seattle, WA, USA).

## 3. Results and Discussion

### 3.1. The Effect of Coffee Cherry Pulp Retention on Physicochemical Quality

#### 3.1.1. Moisture Content and Hardness

**Figure 1:**
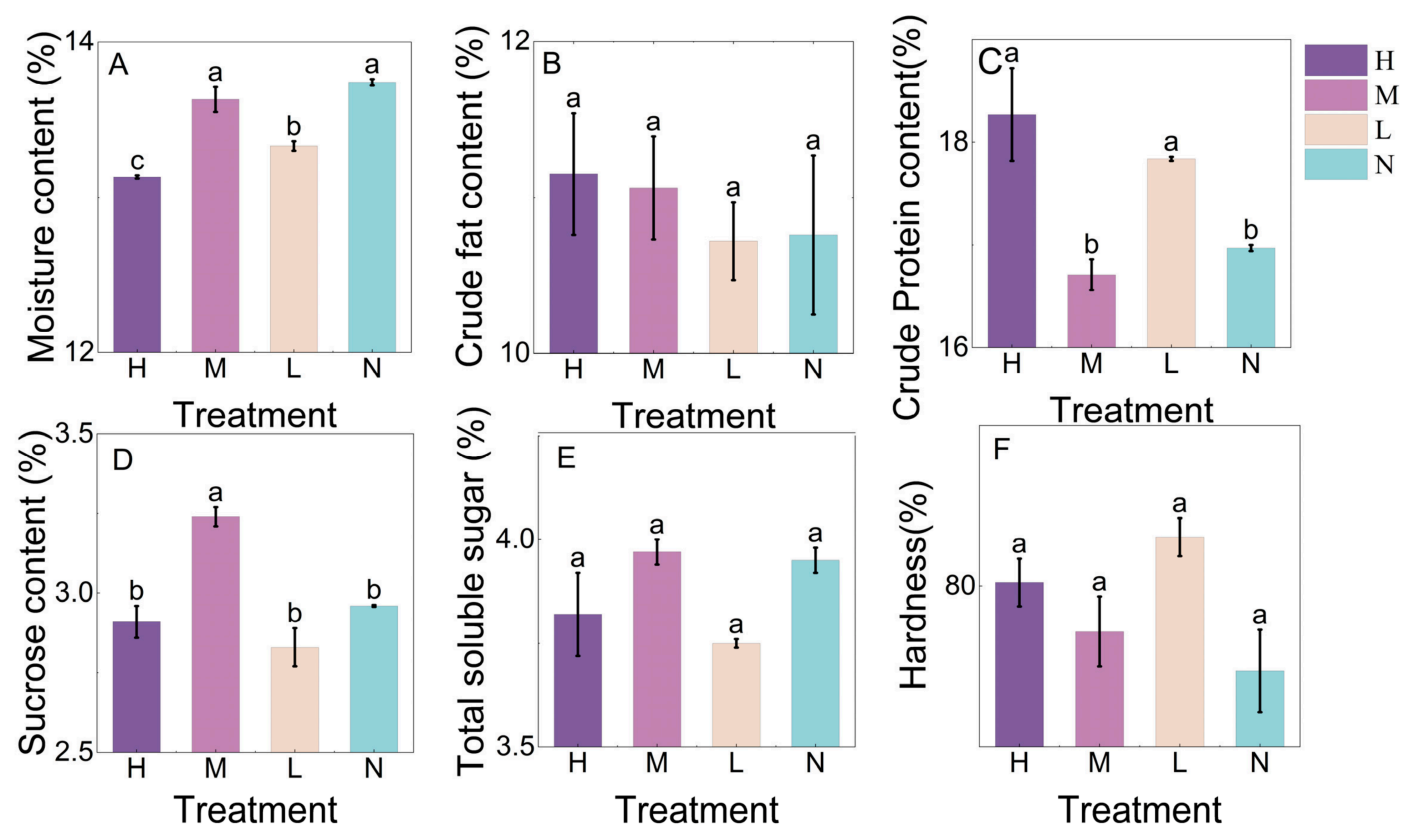
Effect of different coffee cherry pulp retention on various physicochemical quality parameters of coffee samples: (**A**) moisture content; (**B**) crude fat content; (**C**) crude protein content; (**D**) sucrose content; (**E**) total soluble sugars; (**F**) hardness. All data are expressed as mean ± standard error; different lowercase letters indicate significant differences between treatments (*p* < 0.05).

Moisture serves as the core heat reservoir and heat-transfer medium in green coffee beans. The moisture content directly determines the heat storage capacity of the beans, thereby directly influencing the efficiency of heat conduction from the bean surface to the internal endosperm. Moisture, in synergy with temperature, mediates the synthesis, transformation, and degradation metabolism of flavor compounds within green beans, constituting a core physicochemical factor that determines the fundamental quality of green coffee beans and ultimately shapes the final flavor profile of the roasted coffee product. Moisture content of green coffee beans differed significantly among treatments (*p* < 0.05, Figure 1A), with the lowest value observed in the H treatment (13.13%), followed by the L treatment (13.33%). In contrast, the M (13.63%) and N (13.74%) treatments showed significantly higher moisture contents and did not differ significantly from each other. Treatment H had the lowest moisture content, which was significantly lower than that of the N by 4.38%. These results indicate that the relationship between coffee cherry pulp retention and final bean moisture content was nonlinear [1]. A plausible explanation for this nonlinear response is that the coffee cherry pulp matrix may help maintain a relatively loose and porous structure among the beans, thereby potentially facilitating moisture migration. This interpretation is consistent with previous reports that residual pulp can regulate drying kinetics [40], and that different primary processing methods may alter moisture transport by modifying the microporous structure of green coffee beans [1]. However, as only final moisture content was measured and gas exchange dynamics were not monitored during drying, this mechanism remains a hypothesis requiring further validation. However, the effect of coffee cherry pulp retention on final moisture content may depend not only on the amount of retained coffee cherry pulp but also on the physical structure and degradation behavior of the coffee cherry pulp-mucilage layer during fermentation and drying. Although high coffee cherry pulp retention may increase the initial moisture load, prolonged aerobic fermentation, regular turning, and daytime drying may promote coffee cherry pulp degradation and maintain more effective water-diffusion pathways in the H treatment. In contrast, the intermediate coffee cherry pulp-retention level in the M treatment may have formed a relatively compact and viscous coffee cherry pulp-mucilage layer, which could increase resistance to moisture transfer during drying. Fermentation-mediated changes in pectin degradation, microbial activity, and material structure may therefore result in a nonlinear moisture response to coffee cherry pulp-retention level. Nevertheless, as only final moisture content was measured in this study, further investigations involving drying kinetics, water activity, and fermentation parameters are required to verify these mechanisms.

The hardness of green coffee beans is a key physical indicator that characterizes their physical texture and structure, as well as the content of flavor compounds and storage stability [2]. Previous studies have generally confirmed that the moisture content of green coffee beans is significantly negatively correlated with hardness and density. Higher green bean density increases resistance to heat conduction, delays heat transfer rates, and tends to cause quality discrepancies between the surface and interior of the beans. This gradient effect leads to differential accumulation of flavor compounds between the outer and inner regions, ultimately compromising the uniformity of the final roasted coffee product [5]. In this study, varying levels of coffee cherry pulp retention did not significantly alter the density of green coffee beans (Figure 1F), indicating that bean density was not a critical factor mediating the quality differentiation of green coffee beans in our experimental system. Meanwhile, a potential coupling relationship was observed between bean hardness and the storage stability of flavor precursor compounds. Future research will conduct correlation analyses using multiple indicators, including green bean density, color and flavor components, and sensory quality, to systematically elucidate the complete mechanism by which the amount of remaining coffee cherry pulp regulates moisture content and, in turn, influences the overall quality of Robusta green coffee beans.

#### 3.1.2. Crude Protein

Crude protein is the most important nitrogen-containing functional component in green coffee beans. It not only serves as a key physicochemical indicator for evaluating the nutritional level and fruit maturity of green beans, but also acts as a core source of flavor precursors, playing a decisive regulatory role in the physical structure of green coffee beans and the final flavor profile of roasted coffee [6]. The results of this study showed that crude protein contents in green coffee beans of H and treatment L were significantly higher than those of treatment N by 7.11% and 4.86%, respectively (*p* < 0.05, Figure 1C). Coffee cherry pulp retention can reshape the drying kinetics of green beans in the H treatment of green beans and gas exchange conditions within the bean pile microenvironment. By regulating the intracellular protein synthesis and accumulation processes, as well as spatial structural stability, it ultimately induced significant differentiation in crude protein content among different treatments [41]. From the perspective of physical storage, proteins possess excellent hydrophilic gel-forming properties, which allow them to fill the pores in green coffee beans and bind intracellular bound water, thereby enhancing the structural density and mechanical hardness of the beans. This helps mitigate issues such as moisture absorption, swelling, cracking, and mold growth during storage, ensuring the stability of the green coffee beans’ commercial quality [42]. Furthermore, crude protein and its degradation products, including free amino acids, serve as core substrates for the Maillard reaction and Strecker degradation. Differences in protein accumulation among treatments directly alter the abundance of flavor precursors, thereby modulating the generation of heterocyclic characteristic aroma compounds such as pyrazines and pyridines. A negative correlation between protein content and sensory quality was demonstrated in a study of 66 Arabica green coffee bean samples, indicating that higher protein levels reduce the sucrose-to-total titratable acidity ratio, promote the accumulation of bitter compounds, and consequently disrupt the sensory balance of sweetness and acidity in coffee [9]. Taken together, the differences in crude protein content mediated by coffee cherry pulp retention can alter the fundamental quality of green coffee beans through two regulatory pathways: physical structure modification and biochemical substrate supply. Therefore, crude protein serves as a crucial intrinsic factor that differentiates the processing quality and flavor potential of coffee.

#### 3.1.3. Sucrose and Total Soluble Sugars

Sucrose is the most abundant free carbohydrate in Robusta green coffee beans. It serves not only as a key chemical marker for distinguishing varietal genotypes and fruit harvest maturity but also as a crucial substrate for the Caramelization and Maillard reactions during the roasting stage. Its accumulation level directly determines the intensity of sweetness in the finished coffee, the total amount of organic acids produced, and the richness of aromatic compounds, making it a core physicochemical parameter for predicting the flavor potential of green beans during roasting [5,9]. The M treatment had a significantly higher sucrose content (3.24%) than the H, L, and N treatments (*p* < 0.05), whereas no significant differences were found among the latter three treatments. In contrast, total soluble sugar content showed no significant difference among treatments (*p* > 0.05), despite the numerically highest value being observed in the M treatment (Figure 1D,E). Previous studies have suggested that coffee cherry pulp-retention treatments can reshape the internal metabolic flux of green coffee beans, diverting photosynthetic carbon skeletons toward nitrogen-containing metabolic pathways, thereby inhibiting the intracellular accumulation of carbohydrates such as sucrose [43,44]. However, the responses of sucrose and total soluble sugar observed in the present study were not completely consistent. Sucrose is a specific carbohydrate component, whereas total soluble sugar is a composite index that includes sucrose, glucose, fructose, and other soluble carbohydrate fractions. During fermentation and drying, different sugar fractions may be retained, consumed, or converted at different rates. Therefore, the relatively higher sucrose content in the M treatment may have been accompanied by the consumption or transformation of monosaccharides and other soluble sugars, resulting in no significant difference in total soluble sugar content among treatments.

The significantly higher sucrose content in the M treatment suggests that moderate coffee cherry pulp retention, approximately 66% in this study, may provide a relatively favorable environment for sucrose preservation. Moderate coffee cherry pulp retention may maintain a suitable balance between coffee cherry pulp derived substrate availability, microbial activity, and the physicochemical conditions of the bean pile. This interpretation is consistent with previous findings that an appropriate degree of coffee cherry pulp retention can promote the accumulation of sugar-related flavor precursors and improve the quality potential of green coffee beans [19]. In contrast, the lower sucrose content in the H treatment may be associated with more intensive fermentation-related sugar consumption or transformation under high coffee cherry pulp retention. The relatively low sucrose content in the L treatment may be related to limited coffee cherry pulp derived substrates and a less stable fermentation microenvironment. Previous studies have confirmed that sucrose content serves as a key indicator for distinguishing high-quality coffee, with higher sucrose accumulation significantly enhancing coffee sweetness and overall flavor balance [24]. Therefore, the higher sucrose content of the M treatment may enhance the availability of roasting flavor precursors. The results indicate that moderate coffee cherry pulp retention may preferentially promote the retention or redistribution of sucrose, thereby contributing to the differentiation of green coffee bean quality under different primary processing treatments.

#### 3.1.4. Crude Fat

Crude fat is a key component that imparts a rich mouthfeel to coffee. It also serves as an important carrier for volatile aromatic compounds formed during roasting, directly influencing mouthfeel and aroma persistence. Therefore, it is a key physicochemical indicator for evaluating the sensory quality of coffee. The crude fat content of green coffee beans provides a direct indication of the storage stability of lipid components and their response characteristics to the environment during the initial processing of fresh coffee cherries [45]. In this study, no significant differences in crude fat content were observed among the treatments (Figure 1B), indicating that the changes in microenvironmental fermentation and drying conditions induced by coffee cherry pulp-retention treatments did not alter the overall accumulation level of crude fat in green coffee beans. The lipids in green coffee beans primarily exist as triglycerides and diterpene esters. Their strong hydrophobicity effectively impedes the contact between microbial extracellular degradative enzymes and lipid substrates, thereby substantially attenuating lipid degradation during the fermentation process. Previous studies have confirmed that a significant loss of total lipid content occurs only under extreme pretreatment conditions, such as prolonged immersion in water or intense microbial fermentation; fatty acid composition exhibits a nonlinear response to environmental stress, with a decline in content occurring only under severe growth or processing conditions [33,46]. The total crude fat content exhibited high stability across the processing gradients in this study, reflecting the strong processing resistance and genotypic conservatism of total coffee lipids. This finding suggests that quality regulation of coffee under coffee cherry pulp-retention-based primary processing systems need not target total fat content as an optimization endpoint. Instead, research focus should shift toward more refined indicators, such as fatty acid composition profiles and lipid oxidation levels, thereby providing novel targeted strategies for the precise optimization of differentiated processing technologies for fresh coffee cherries.

### 3.2. The Effect of Coffee Cherry Pulp Retention on Bioactive Compounds

**Figure 2:**
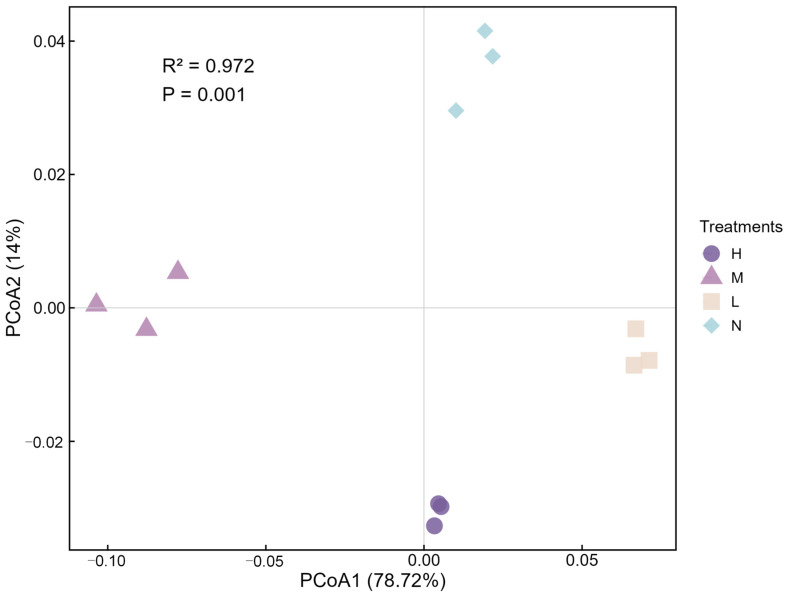
PCoA of coffee samples under four coffee cherry pulp-retention treatments based on physicochemical and functional activity indicators. Colors indicate treatments: H, M, L, and N.

The PCoA analysis further corroborated the above differentiation at the overall compositional level. PC1 (78.72%) and PC2 (14%) together accounted for 92.72% of the total variance (Figure 2), and the four coffee cherry pulp-retention treatments were clearly separated in the ordination space, indicating that coffee cherry pulp retention does not merely alter individual components but is associated with an overall shift in the compositional profile of green coffee beans. Notably, treatment M was distinctly separated from H, L, and N, rather than occupying an intermediate position between the L and H treatments. This distribution suggests that the response to coffee cherry pulp retention was nonlinear, with the intermediate retention level producing a unique compositional profile. H was characterized by relatively higher alkaloid- and protein-related characteristics, whereas M showed relatively higher organic acid- and sugar-precursor characteristics, and L exhibited relatively higher levels of polyphenols and flavonoids. These clustering results further suggest the notion that the amount of coffee cherry pulp retention shapes distinct compositional characteristics, possibly through differences in the fermentation environment, substrate availability, and redox status [47,48]. In particular, the distinct position of M is consistent with its superior comprehensive quality and higher sucrose content, suggesting that moderate coffee cherry pulp retention may provide a more favorable balance between substrate supply and fermentation intensity. However, because microbial succession, pH, redox status, and dynamic metabolic changes were not monitored in the present study, the mechanisms underlying this nonlinear response require further verification. Metabolomic data from naturally processed and washed coffee beans were analyzed using PCoA, and it was similarly observed that different processing conditions drove structured, processing-specific reorganization of metabolic fingerprints, further confirming the systematic reshaping of the coffee chemical profile by the processing environment [25].

#### 3.2.1. Total Alkaloids

**Figure 3:**
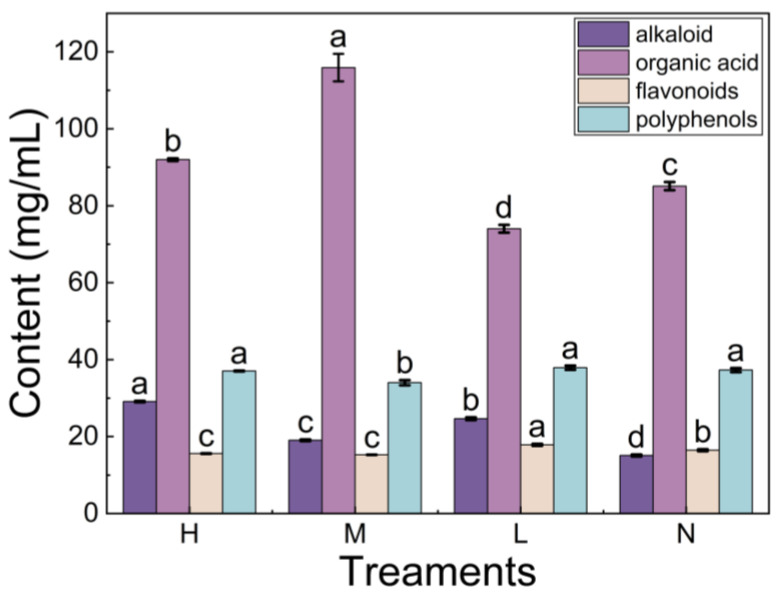
Effects of different treatments (H, M, L, and N) on the contents of alkaloids, organic acids, flavonoids, and polyphenols in coffee samples. Different lowercase letters indicate significant differences between treatments (*p* < 0.05).

Alkaloids serve as the core bitter taste compounds in coffee and also act as important precursors for heterocyclic aroma components. Their intracellular metabolic homeostasis directly determines the sensory flavor profile of coffee. The total alkaloid level can reflect the plant nitrogen allocation flux under the coffee cherry pulp-retention-induced microenvironment, the fermentation intensity of fresh cherries, as well as the structural differentiation of microbial communities and secondary metabolic pathways within the system [16]. In this study, the total alkaloid contents in H, M, and L were significantly higher than those in N by 48.15%, 20.72%, and 38.74%, respectively (*p* < 0.05, Figure 3), suggesting that high coffee cherry pulp retention significantly activates nitrogen secondary metabolism and promotes the continuous enrichment of nitrogenous alkaloid compounds. Previous studies comparing natural and washed processing methods have similarly confirmed that varying levels of coffee cherry pulp retention can significantly alter the alkaloid accumulation in green coffee beans [49]. This finding corroborates the patterns of alkaloid differentiation induced by different coffee cherry pulp-retention gradients observed in this study, highlighting that the fermentation microenvironment is a key factor in regulating alkaloid metabolism. From the perspective of coffee flavor development, an appropriate content of alkaloids not only establishes a rich, bitter foundation for the coffee but also, through degradation, generates signature heterocyclic aromatic compounds such as pyrazines and pyridines, thereby enriching the aromatic complexity of the final product. In summary, coffee cherry pulp retention can regulate the balance between alkaloid biosynthesis and degradation by reshaping nitrogen metabolism and microbial fermentation dynamics, synergistically optimizing both the bitter taste characteristics of coffee and the reserve levels of roasting aroma precursors.

A total of 55 alkaloid compounds were identified in this study. Previous studies have confirmed that caffeine and trigonelline are the predominant constituents and key flavor precursors of total alkaloids in green coffee beans, with their combined levels largely determining the total alkaloid content. They are recognized as hallmark physicochemical indicators for evaluating genotypic differences, fruit maturity, and intrinsic flavor potential. Functionally, caffeine primarily contributes to the intense bitterness and astringent mouthfeel of coffee, while also participating in secondary metabolism related to plant defense against insect pests. Trigonelline, on the other hand, exhibits high flavor precursor conversion potential, decomposing to generate heterocyclic aroma compounds and nicotinic acid, thereby simultaneously determining the aroma complexity and nutritional value of green coffee beans. Quantitative results showed that caffeine contents in M and L were significantly higher than those in N by 23.44% and 29.16%, respectively. Moreover, caffeine content in L was also significantly higher than in H by 17.97% (*p* < 0.05, Table 1). The trigonelline content in treatment N was 17.08% lower than that in L (Table 1). This reduction may be attributed to the extensive leaching loss of water-soluble nitrogenous flavor precursors, including caffeine and trigonelline, during the soaking process involved in the N treatment. In contrast, the retention of pectic polysaccharides in the cherry pulp provides sufficient carbon and nitrogen substrates for the metabolism of microbial communities associated with green coffee beans. By reshaping intracellular signaling pathways, this substrate availability continuously activates alkaloid biosynthesis, ultimately driving the significant enrichment of caffeine and trigonelline observed in M and L [50]. Notably, the intensive fermentation environment formed by full coffee cherry pulp retention in H contained sufficient metabolic substrates. However, excessive alkaloids were decomposed and utilized by microorganisms as nitrogen sources to support their proliferation and trigger directional metabolic transformation, ultimately leading to lower caffeine accumulation in H than in L.

#### 3.2.2. Total Organic Acids

Total organic acids serve as a comprehensive core indicator of the acid profile, flavor precursor levels, and post-harvest microbial metabolic activity in green coffee beans. They can accurately characterize the dynamic metabolic balance between carboxylic acid accumulation and phenolic acid degradation during post-harvest processing, and objectively reflect the metabolic steady-state characteristics of flavor precursors in green coffee beans [1,51]. Post-harvest coffee cherry pulp-retention-induced microenvironmental changes can modulate microbial metabolic activity and intracellular enzymatic conversion efficiency, thereby reshaping the accumulation patterns of organic acids and phenolic compounds in green beans, ultimately mediating the differentiation of coffee flavor quality. The results of this study showed that total organic acid content in H and M was increased by 36.20% and 8.08%, respectively, compared with N, while that in L was significantly decreased by 13.01% (*p* < 0.05, Figure 3). Furthermore, chlorogenic acid (CGA) content in N was significantly reduced by 25.61% compared with L (*p* < 0.05, Table 1). Based on established knowledge that lactic acid bacteria can metabolize sucrose to organic acids via glycolysis and heterolactic fermentation [52], we hypothesize that moderate pulp retention may create favorable conditions for LAB proliferation, potentially contributing to the observed organic acid enrichment in the M treatment. However, because pH, oxygen levels, and microbial dynamics during fermentation were not directly measured, this interpretation remains a hypothesis rather than a confirmed pathway. By contrast, the low-water-activity microenvironment associated with high coffee cherry pulp retention in L effectively attenuates the oxidative degradation activities of polyphenol oxidase (PPO) and peroxidase (POD), thereby minimizing oxidative polymerization losses of chlorogenic acid and other phenolic acids, and preserving phenolic functional compounds. Nevertheless, excessive coffee cherry pulp retention may trigger overfermentation, intensify competition for metabolic substrates, and promote excessive metabolite conversion, ultimately constraining the effective accumulation of flavor precursors and compromising overall coffee quality.

Previous studies have successfully isolated and identified LAB and yeasts during the drying process of fresh coffee cherries, reporting that these microbial populations can increase the contents of organic acids such as quinic acid and lactic acid in green beans [16,53]. Furthermore, the selective enzymatic hydrolysis of chlorogenic acid can effectively reduce the accumulation of bitter compounds in coffee, thereby optimizing its flavor quality. As core substrates for esterification reactions, carboxylic acids drive the synthesis of high-quality volatile aromatic compounds such as fruity and wine-like aromas; meanwhile, caffeic acid and quinic acid, which are generated through phenolic acid metabolism, serve not only as important precursors for characteristic phenolic aromas during roasting but also as key components that regulate the perception of coffee bitterness and balance the overall acid profile, collectively forming the material basis for the expression of coffee’s acidity and aroma [15]. Coffee cherry pulp-retention treatments dynamically optimize coffee acidity characteristics and flavor potential through the synergistic regulation of carboxylic acid metabolism and phenolic acid degradation. In summary, different gradient levels of coffee cherry pulp retention can differentially modulate microbial fermentation metabolism and enzymatic oxidative reactions, orchestrating a dynamic balance between carboxylic acid accumulation and phenolic acid degradation. This approach enables precise optimization of the acidic structure and flavor reserve potential of green coffee beans, representing a key processing strategy for regulating functional compound accumulation and quality formation in coffee.

#### 3.2.3. Total Polyphenols

Total polyphenols are core functional metabolites that determine the in vitro antioxidant activity and flavor formation potential of green coffee beans. Their accumulation level and metabolic homeostasis not only define the nutritional and functional value of green coffee beans but also serve as pivotal metabolic nodes linking green bean quality characteristics. The results of this study indicated that the total polyphenol content in M was significantly decreased by 8.77% compared with N (*p* < 0.05, Figure 3). The total polyphenol contents in L and H were higher than those in N, indicating that only moderate coffee cherry pulp retention caused significant polyphenol loss, while both low retention and excessive retention did not produce obvious negative effects. It is plausible that the high-moisture, high-oxygen microenvironment associated with high pulp retention may activate PPO and POD activities, potentially accelerating phenolic oxidation [54]. In contrast, in the low coffee cherry pulp retention (L), the processes of water penetration and oxygen diffusion are restricted, which can significantly reduce the intensity of both non-enzymatic and enzymatic oxidation reactions and effectively mitigate the degradation and loss of polyphenols. Thus, the coffee cherry pulp-retention gradient can differentially regulate the metabolic balance between the synthesis and degradation of phenolic compounds by reshaping the water activity and oxygen mass transfer efficiency within the soybean pile [55]. Among all treatments, the low-moisture microenvironment established in L exhibited the most favorable effect on the stable retention of polyphenolic components. In summary, the regulatory effect of coffee cherry pulp retention on total polyphenol accumulation in green coffee beans depends on the dynamic balance between phenolic synthesis rate and oxidative consumption. The microenvironmental conditions in M disrupted this balance, resulting in a significant reduction in polyphenol content. In contrast, H relied on abundant carbon and nitrogen substrates to continuously supply precursors for polyphenol synthesis, while L depended on low moisture to inhibit oxidative enzyme activities. These two distinct pathways both effectively maintained phenolic metabolic homeostasis and achieved stable polyphenol enrichment.

#### 3.2.4. Total Flavonoids

Total flavonoids serve as the core functional components responsible for the antioxidant activity of green coffee beans. They not only determine the mouthfeel intensity and flavor aroma retention duration of coffee but also serve as a key indicator for balancing the sensory flavor and bioactivity value of green coffee beans [56]. The results of this study showed that total flavonoid contents in H and M were significantly reduced by 5.24% and 7.18%, respectively, compared with N, while total flavonoid content in L was significantly increased by 7.87% relative to N (*p* < 0.05, Figure 3). These findings indicate that the intracellular accumulation of flavonoids is highly sensitive to the level of coffee cherry pulp retention, and efficient flavonoid enrichment can only be achieved within an optimal range of coffee cherry pulp retention. It is possible that the lower moisture and oxygen availability in the L treatment limited PPO and POD activities, thereby reducing flavonoid oxidation. This interpretation is consistent with previous findings on phenolic oxidation in olive oil [57]. In comparison, H and M constructed a fermentation microenvironment with high moisture and vigorous oxygen exchange, which readily triggered oxidative polymerization of flavonoid monomers and markedly reduced the content of extractable total flavonoids. Previous studies on extra virgin olive oil have verified a significant positive correlation between system water content and phenolic oxidative loss, which supports the conclusion of the present study that high-moisture microenvironments accelerate flavonoid degradation during coffee fermentation [57]. In conclusion, low coffee cherry pulp retention (L) establishes a dynamic balance between microbial fermentation intensity and flavonoid oxidative loss, serving as a critical processing condition for maximizing the net accumulation of flavonoids in green coffee beans. The low oxidative-stress microenvironment in L effectively alleviates flavonoid degradation, resulting in a significantly higher flavonoid accumulation capacity compared with the coffee cherry pulp-free washed control treatment. This treatment endows green coffee beans with superior in vitro antioxidant activity and enriches the reservoir of flavor precursor components.

### 3.3. Bacterial Community Composition Under Different Coffee Cherry Pulp-Retention Levels

**Figure 4:**
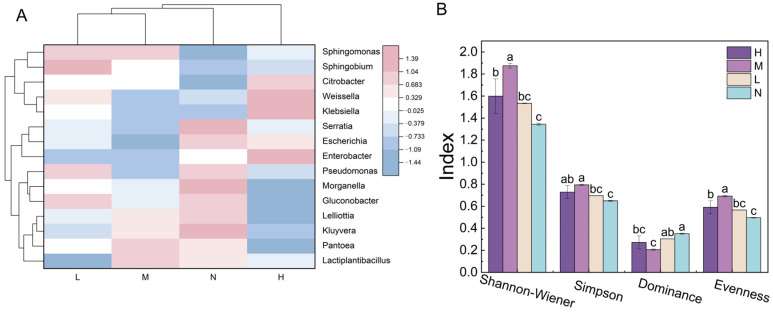
Bacterial community composition and alpha diversity under different coffee cherry pulp-retention levels. (**A**) Heatmap of dominant bacterial genera across treatments. (**B**) Shannon–Wiener, Simpson, dominance, and evenness indices. Different lowercase letters indicate significant differences among treatments (*p* < 0.05).

The genus-level heatmap showed that the relative-abundance patterns of dominant bacterial genera differed among the four treatments (Figure 4A). Pantoea, Enterobacter, and Klebsiella were the dominant genera across all four treatments, although their relative contributions varied with coffee cherry pulp-retention rate. In the H treatment, Pantoea and Enterobacter were the predominant genera associated with this treatment. The M treatment showed relatively high abundances of Sphingobium, Citrobacter, Weissella, Klebsiella, Serratia, and Enterobacter. The L treatment exhibited higher relative contributions from Klebsiella and Enterobacter, whereas the N treatment was predominantly characterized by Enterobacter, accompanied by several low-abundance genera. Hierarchical clustering indicated that the H and N treatments had relatively similar bacterial compositional profiles, whereas the L and M treatments showed distinct distribution patterns. These results indicate that coffee cherry pulp retention altered the compositional distribution of the fermentation bacterial community at the genus level, and this shift did not follow a simple linear trend with increasing retention rate. Alpha diversity analysis further showed significant differences among treatments. The M treatment exhibited the highest Shannon–Wiener index, Simpson index, and evenness, as well as the lowest dominance index, indicating that it had the highest bacterial community diversity and the most even distribution of taxa. In contrast, the L treatment showed the lowest Shannon–Wiener index and Simpson index, together with a relatively high dominance index, suggesting lower community diversity and a greater dominance of certain taxa (Figure 4B).

The observed compositional shifts may be related to differences in substrate availability, moisture, and oxygen conditions created by varying pulp-retention levels. For example, higher pulp retention provides more fermentable substrates, which may favor genera such as Pantoea. In contrast, lower coffee cherry pulp retention may create different substrate concentrations and mass-transfer conditions, thereby allowing Klebsiella and Enterobacter to occupy larger proportions. Notably, the M treatment was characterized by a consortium of multiple dominant genera rather than being highly dominated by a single genus. The M treatment also exhibited higher contents of sucrose, organic acids, esters, and other flavor-related components, and received the highest comprehensive quality evaluation. A possible interpretation is that approximately 66% pulp retention may establish a more suitable balance among substrate supply, microbial metabolism, and fermentation intensity. The higher bacterial diversity and evenness observed in the M treatment may have contributed to a broader range of microbial metabolic activities, thereby supporting the accumulation of flavor-related compounds. However, the heatmap and alpha diversity indices reflect compositional and diversity differences only and do not establish a direct causal link between specific genera and quality changes. Therefore, these results suggest that moderate coffee cherry pulp retention may promote Robusta coffee quality formation by modulating the composition of the fermentation microbial community, although the underlying causal mechanisms still require further elucidation through differential abundance analysis, genus–metabolite correlation analysis, and strain-level functional validation.

### 3.4. The Effect of Coffee Cherry Pulp Retention on Volatile Flavor Precursor Compounds

**Figure 5:**
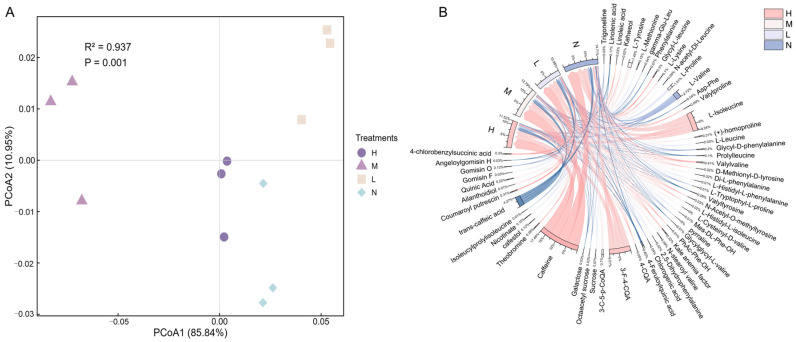
PCoA based on physicochemical and functional activity indicators (**A**) and metabolite association analysis of coffee beans under different coffee cherry pulp-retention treatments (**B**).

In this study, a total of 551 volatile flavor precursor compounds were identified in green coffee beans, belonging to ten chemical families: pyridines, pyrroles, pyrazines, alcohols, phenols, furans, aldehydes, acids, indoles, and esters. Volatile precursors present in green beans, such as alcohols, phenols, and pyrroles, serve as key substrates for aroma formation during roasting and directly determine coffee flavor potential and quality [58]. Different coffee cherry pulp-retention treatments had a significant impact on the composition of volatile precursors (Figure 5B), indicating that the post-harvest processing stage can exert a prescriptive influence on subsequent roasting aroma potential. PCoA results showed that the volatile precursor profiles of different treatments exhibited a distinct separation pattern, with M displaying particularly unique metabolic characteristics that clearly distinguished it from the other three treatments; the first two principal coordinates of PCoA cumulatively explained 96.79% of the total variation (PCoA1: 85.84%, PCoA2: 10.95%, Figure 5A), fully reflecting the overall pattern of differences among the samples [35,59]. M was the farthest from the other three treatments, indicating the most significant separation in community composition or metabolic characteristics. Meanwhile, the other three treatments largely overlapped on PCoA1 and differed only on PCoA2, suggesting that they share similar secondary features, with major differences primarily driven by the factors represented by the first dimension. This differentiation pattern may be related to the differential availability of fermentable substrates, moisture, and oxygen conditions created by varying pulp-retention levels, as well as potential differences in the physicochemical microenvironment during drying [60].

**Figure 6:**
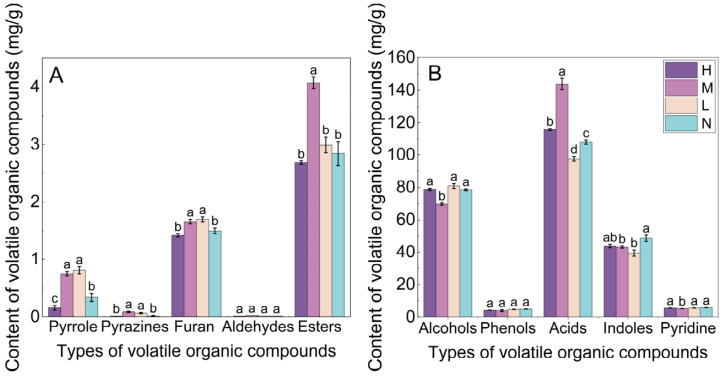
Effects of coffee cherry pulp-retention treatments on volatile organic compounds in coffee. (**A**) Content of pyrrole, pyrazines, furan, aldehydes, and esters; (**B**) Content of alcohols, phenols, acids, indoles, and pyridine. Different lowercase letters indicate significant differences among treatments (*p* < 0.05).

By category, alcohols, pyrroles, pyrazines, and esters were found in higher contents in M and L, indicating that moderate or low coffee cherry pulp retention promotes the accumulation of certain aroma precursors; in contrast, phenols and indoles were enriched in N, suggesting that the washed treatment may have resulted in a precursor composition distinct from that of the coffee cherry pulp-retention treatments (Figure 6). The uniqueness of the flavor precursor profile in M may stem from its moderate microbial metabolic activity: moderate coffee cherry pulp retention provides an adequate supply of substrates while preventing the excessive consumption of key sugars and amino acids caused by overfermentation, thereby promoting the formation of alcohol, ester, and nitrogen-containing heterocyclic precursors. Esters are typically associated with fruity and fermented aromas; alcohols serve as substrates for esterification reactions; and pyrazines and pyrroles are important sources of nutty, roasted, and burnt aromas during the roasting process. The relative enrichment of these compounds in M indicates that moderate coffee cherry pulp retention offers the advantage of enhancing aroma complexity and the potential for roasted aromas. Single-bean volatiles of Arabica and Robusta coffees from 13 origins were analyzed, and it was found that green bean precursor composition contributed even more to final volatile profile variation than roast degree, further confirming the importance of post-harvest precursor regulation [58]. In a comparative study of hot and cold brew coffees, pyrazines were identified as the primary contributors to coffee aroma [34], which aligns with our finding of pyrazine enrichment in M and L, validating the role of pyrazines as key roasting aroma precursors and highlighting the regulatory advantage of moderate coffee cherry pulp retention in their accumulation. In summary, coffee cherry pulp-retention treatments, through differential regulation of volatile precursor composition and metabolic accumulation in green beans, provide essential experimental evidence for the targeted optimization of green coffee quality and the precise design of post-harvest processing parameters.

### 3.5. Grey Relational Analysis and Comprehensive Quality Evaluation

Grey relational analysis (GRA) provided quantitative criteria for evaluating the quality differences among different coffee cherry pulp-retention treatments from a multi-indicator comprehensive evaluation perspective. The results showed that M exhibited the optimal overall quality, and equal-weight analysis further confirmed the robustness of this ranking (Table 2). The comprehensive quality of M was improved by 6.057%, 18.51%, and 13.74% compared with L, H, and N, respectively, indicating that moderate coffee cherry pulp retention achieves a superior balance among functional components (Table 3), flavor precursors, and fundamental physicochemical indicators. In contrast to single-indicator comparisons, GRA emphasizes the overall proximity of multiple dimensions to an ideal quality state, thus more comprehensively reflecting the integrated regulatory effects of processing methods on green coffee bean quality. The superior quality of M was mainly attributed to the synergistic accumulation of sucrose, organic acids, and volatile precursors. Moderate coffee cherry pulp retention maintained sufficient carbohydrate substrate supply, promoted the accumulation of organic acids and the directional generation of aroma precursors, and consequently established a coordinated balance among carbon metabolism, microbial fermentation, and flavor formation. In comparison, L contained high levels of polyphenols and flavonoids but suffered from insufficient organic acid supply, while H exhibited relatively high alkaloid and protein contents but lacked sucrose and organic acids, resulting in imbalanced quality profiles in both treatments. Under the experimental conditions in this study, the coffee cherry pulp-retention level corresponding to M was therefore identified as the optimal treatment (Table 2). Nevertheless, further verification based on multi-batch and multi-region trials together with sensory evaluation is still required to determine the universal optimal coffee cherry pulp-retention ratio. In summary, moderate coffee cherry pulp retention was associated with the concurrent accumulation of sugars, organic acids, and volatile precursors. We hypothesize that this response may be related to a more favorable balance of drying and fermentation conditions, which could influence substrate availability, carbon and nitrogen allocation, and phenolic oxidation during processing. This study provides experimental evidence and theoretical support for the precise optimization of post-harvest processing parameters and targeted quality regulation of Robusta coffee.

### 3.6. Effect of Different Levels of Coffee Cherry Pulp Retention on the Sensory Quality of Robusta Coffee

**Figure 7:**
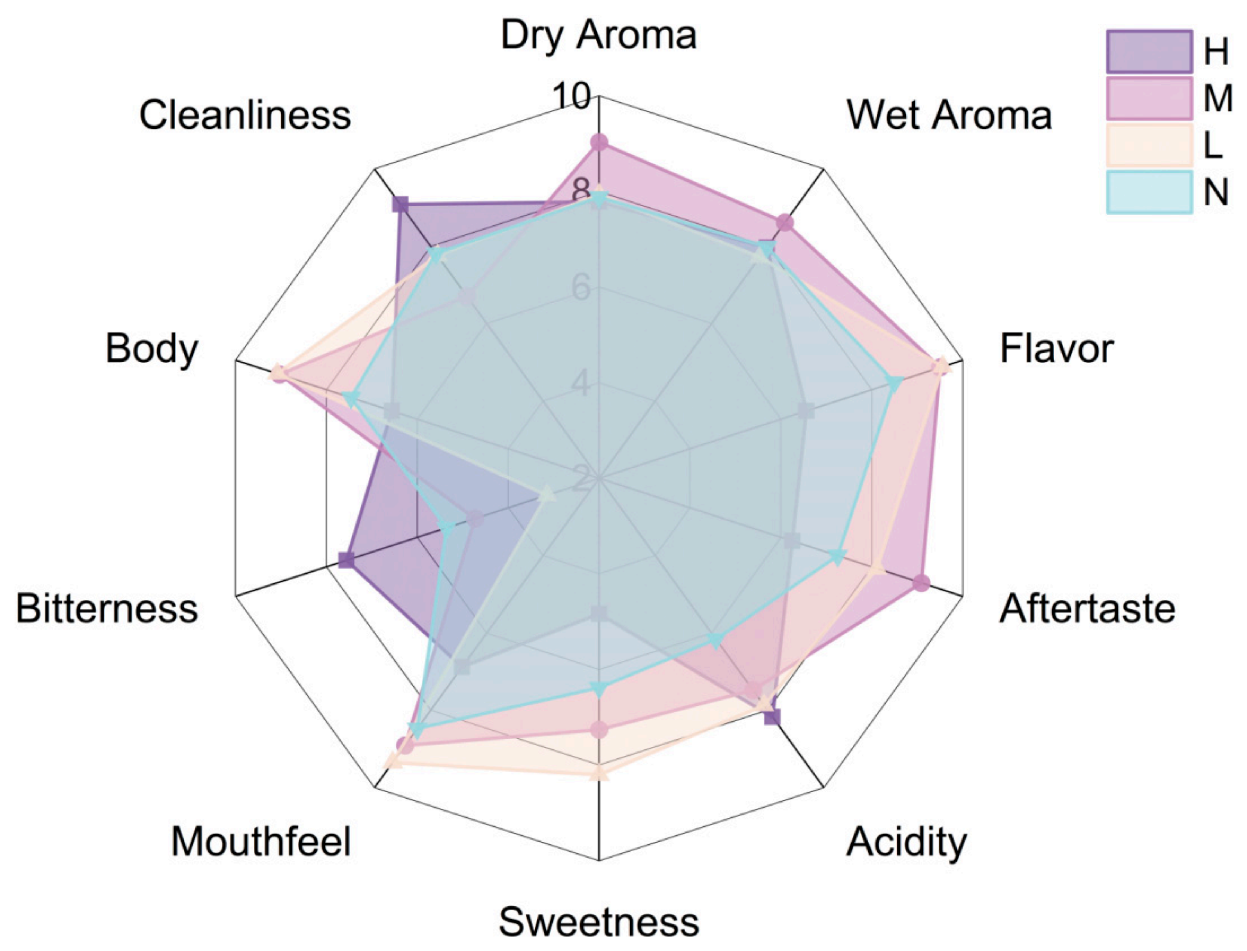
A radar chart showing the sensory attributes of Robusta coffee under different levels of pulp retention. Sensory attributes include dry aroma, wet aroma, flavor, aftertaste, acidity, sweetness, mouthfeel, bitterness, body and cleanliness.

To further verify the impact of the aforementioned changes in physicochemical indicators, bioactive compounds, and volatile flavor precursors on the final quality of the coffee, sensory evaluations were conducted on samples treated with different levels of coffee cherry pulp retention. The results are shown in Table 4 and Figure 7. There were significant differences in the overall sensory quality among the different treatment samples, with the following order of average total scores: 66% treatment (80.34 points) > 33% treatment (78.71 points) > 100% treatment (73.73 points) > 0% treatment (71.65 points). Among these, the treatment with 66% coffee cherry pulp retention exhibited the best overall sensory quality, ranking first with improvements of 2.02%, 10.83%, and 8.84% compared to the 33%, 100%, and 0% treatments, respectively. This indicates that moderate retention of coffee pulp can significantly improve the cupping quality of Robusta coffee.

Looking at individual sensory attributes, the 66% treatment received the highest scores in dry aroma (9.03 points), wet aroma (8.61 points), flavor (9.50 points), and aftertaste (8.11 points), while its mouthfeel (8.91 points) and body (9.03 points) were also at high levels, indicating that this treatment promotes the development of more intense, rich, and persistent aroma and flavor characteristics. In contrast, the 33% treatment performed better in terms of acidity, sweetness, mouthfeel, and body, with scores of 7.87, 8.20, 9.35, and 3.15, respectively, indicating that a lower percentage of retained fruit skin helps enhance the coffee’s acidity, sweetness, and mouthfeel; however, its scores for dry aroma, wet aroma, and aftertaste were all lower than those of the 66% treatment, so its overall color quality was inferior to that of the 66% treatment. Combining the aforementioned physicochemical indicators with the analysis results of volatile flavor precursors, the 66% treatment exhibited higher levels of flavor-related compounds such as sucrose, organic acids, pyrazines, and esters. The abundant sugars and organic acids in the retained pulp provide carbon sources and metabolic substrates for fermentation microorganisms, promoting enzymatic reactions and the transformation of flavor compounds. Among these, sugars and amino acids can participate in the Maillard reaction during roasting, promoting the formation of characteristic flavors such as caramel, nutty, and roasted notes, while organic acids and esters help enhance the balance between acidity and sweetness as well as the aromatic complexity of the coffee. Retaining an appropriate amount of pulp may also help maintain the stability of the fermentation system, inhibit the excessive proliferation of undesirable microorganisms, and thereby improve the final cupping quality.

### 3.7. The Effect of Coffee Cherry Pulp Retention on Volatile Flavor Precursor Compounds Investigated via Co-Occurrence Network Analysis

**Figure 8:**
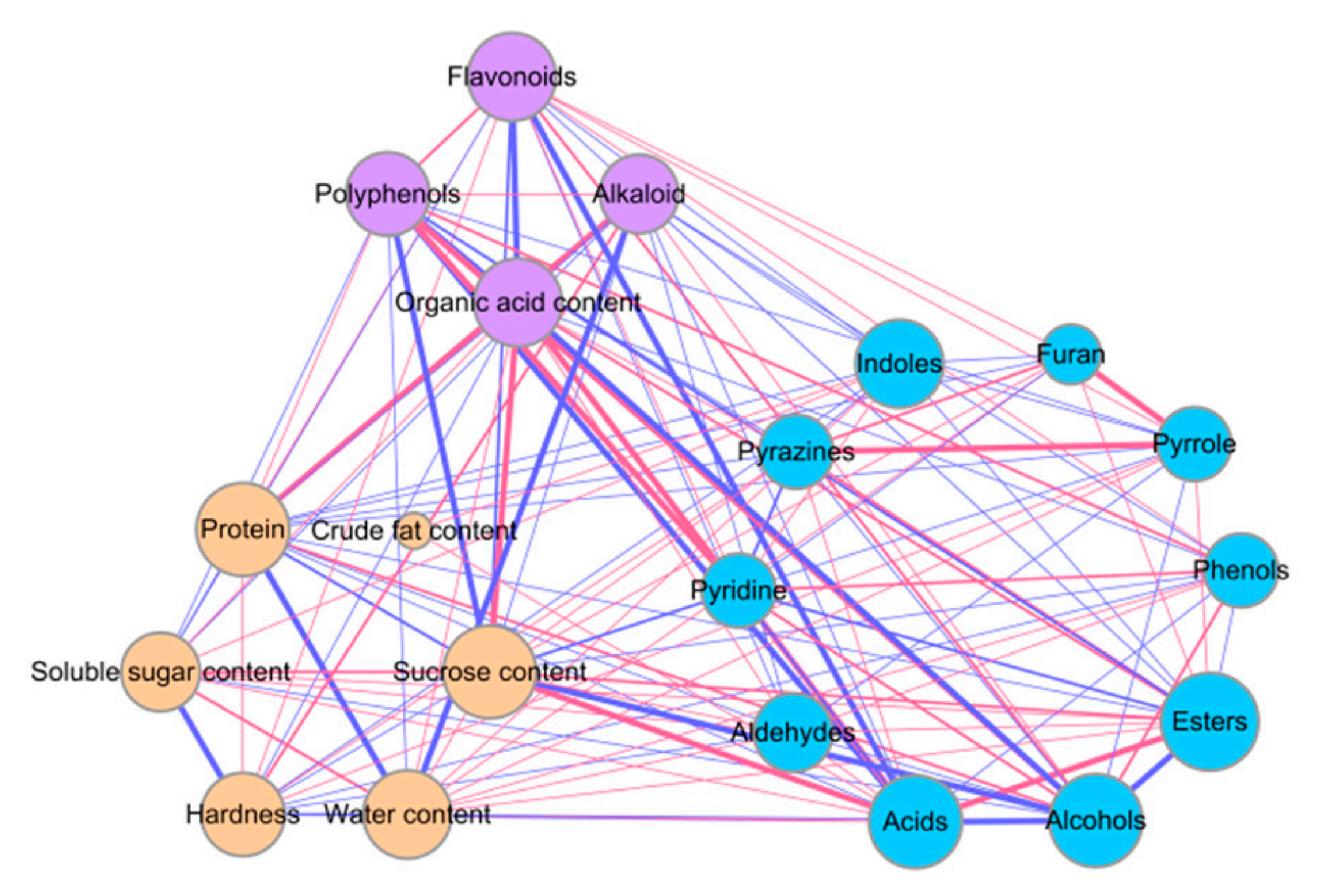
Correlation network of physicochemical, nutritional, bioactive, and volatile flavor-related indicators in green coffee beans under different coffee cherry pulp-retention treatments. Node colors represent different indicator categories: purple nodes indicate functional components, orange nodes indicate physicochemical quality, and cyan nodes indicate volatile flavor precursor compounds. The line colors represent positive (red lines) and negative (blue lines) correlations.

Through network analysis, this study identified co-occurrence patterns among functional bioactive compounds, nutritional and textural components, and volatile flavor precursor compounds (Figure 8, Table 5). Overall, both positive and negative correlations coexist in the entire network. Notably, nutritional components exhibit a significant negative co-occurrence relationship with functional bioactive compounds and a significant positive co-occurrence relationship with volatile flavor precursor compounds, indicating that both synergistic accumulation and metabolic competition exist among different quality indicators. Notably, under different coffee cherry pulp-retention treatments, the negative correlations between alkaloids and esters, pyridines, phenols, and sucrose were particularly significant, suggesting that excessive alkaloid accumulation may inhibit the formation of fruity esters and mild phenolic compounds during roasting, thereby compromising green coffee bean quality. In contrast, organic acids displayed more pronounced positive correlations with proteins, alkaloids, and sucrose, indicating that sucrose can continuously supply carbon skeletons for organic acid biosynthesis, while free amino acids derived from protein hydrolysis may synergistically activate purine alkaloid biosynthetic pathways together with organic acids, thereby enriching the core flavor precursor pool of coffee [61]. Overall, different treatments did not alter the fundamental correlation framework among quality indicators, but did modify the strength of associations among certain key indicators. Across treatments, a general pattern emerged in which nutrients provide the foundational basis, functional components participate in regulatory roles, and volatile compounds determine flavor expression. The M represents a metabolically balanced processing approach, in which functional active components, nutritional–textural foundations, and volatile flavor precursor compounds are coordinated in both intensity and proportion, ensuring simultaneous optimization of functional compound enrichment and flavor potential reserve.

### 3.8. Limitations of the Study and Future Research Directions

This study has several limitations. The experimental samples were collected from a single origin in Wanning, Hainan, which may limit the geographical generalizability of the findings. Although all treatments were processed simultaneously at the same experimental site under an identical daily schedule to minimize environmental and handling variation, further validation is needed across different production areas, harvest seasons, coffee varieties, and processing conditions. Additionally, although the discussion proposes potential mechanisms involving microbial activity, fermentation intensity, oxygen transfer, and redox conditions, direct measurements of pH, oxygen levels, microbial succession, fermentation metabolites, or drying kinetics were not conducted during the process. Consequently, these mechanistic interpretations should be viewed as working hypotheses requiring further validation through targeted fermentation monitoring and controlled experiments.

Future research should conduct controlled fermentation experiments with real-time monitoring of pH, oxygen levels, microbial succession, and fermentation metabolites to validate the mechanistic hypotheses proposed in this study. The effects of coffee cherry pulp retention on the fermentation environment, microbial communities, drying kinetics, and metabolic changes in green coffee beans also warrant further investigation. Integrating multi-omics approaches, such as metabolomics and transcriptomics, may help elucidate the molecular regulatory networks through which processing affects coffee quality. Roasting trials and volatile-compound analyses should also be combined with sensory and consumer evaluations to further verify the effects of pulp retention on roasted coffee quality. In addition, practical factors, including drying efficiency, microbial safety, processing cost, and commercial-scale feasibility, should be assessed to support broader application.

## 4. Conclusions

The different coffee cherry pulp-retention gradients significantly alter the accumulation levels of nutritional components, bioactive compounds, and volatile flavor precursor compounds in Robusta green coffee beans. Overall, the coffee cherry pulp-retention treatments showed higher accumulation of bioactive compounds such as polyphenols and alkaloids, as well as characteristic aroma precursors, compared to the coffee cherry pulp-free control treatment; specifically, the 66% coffee cherry pulp-retention treatment simultaneously enriched key roasting flavor precursors such as sucrose, organic acids, pyrazines, and esters, while significantly reducing the content of components such as proteins, alkaloids, and flavonoids. The quality of Robusta green coffee beans is significantly superior to that of other treatments by 6.05–18.51% under grey relational analysis. This study clarifies the optimal degree of coffee cherry pulp retention for post-harvest processing of Robusta coffee, providing theoretical support and practical guidance for coordinating the nutritional reserves, antioxidant activity, and roasting aroma potential of green beans, thereby enabling the targeted optimization of primary processing methods.

## Figures and Tables

**Table 1:** Effects of different coffee cherry pulp-retention treatments on the content of key compounds.

Category	Name	Treatments			
		H	M	L	N
Flavor	Caffeine	20,898.63 ^ab^	23,574.53 ^a^	25,478.16 ^a^	18,048.69 ^b^
	Theobromine	194.97 ^b^	142.63 ^b^	586.50 ^a^	486.35 ^a^
	Trigonelline	27.72 ^c^	40.87 ^b^	55.50 ^a^	17.32 ^c^
	Chlorogenic acid	45.10 ^b^	30.23 ^c^	70.77 ^a^	52.64 ^b^
	Isochlorogenic acid	58.41 ^b^	45.49 ^c^	55.47 ^b^	68.69 ^a^
	Quinic acid	1408.32 ^b^	1456.19 ^b^	2500.32 ^a^	1441.28 ^b^
	Ferulic acid	10,881.64 ^bc^	10,788.09 ^c^	12,445.00 ^a^	11,236.44 ^b^
	Succinic acid	373.50 ^b^	360.56 ^b^	379.56 ^ab^	397.33 ^a^
	Caffeic acid	5662.42 ^a^	4826.99 ^c^	5621.94 ^a^	5400.05 ^b^
	Niacin	188.20 ^ab^	170.81 ^b^	182.02 ^ab^	204.13 ^a^
	Tannins	412.81 ^c^	432.38 ^c^	639.56 ^a^	505.97 ^b^
Fragrant	Methionine	81.08 ^b^	449.77 ^a^	166.20 ^b^	57.99 ^b^
	Leucine	200.67 ^c^	1865.49 ^a^	633.86 ^b^	229.63 ^c^
	Proline	1637.34 ^a^	1886.58 ^a^	1683.42 ^a^	1513.03 ^a^
	Isoleucine	9956.38 ^ab^	14,156.85 ^a^	6511.70 ^b^	9878.58 ^ab^
	Phenylalanine	238.79 ^d^	990.30 ^a^	439.03 ^b^	316.68 ^c^
	Valine	3020.18 ^b^	8101.65 ^a^	3387.94 ^b^	2673.64 ^b^
	Tyrosine	1204.18 ^c^	3132.59 ^a^	2221.70 ^b^	1177.76 ^c^
	Cysteine	376.27 ^ab^	346.08 ^b^	435.09 ^a^	430.42 ^a^
	Lysine	79.16 ^c^	252.51 ^a^	118.18 ^b^	59.42 ^d^
	Glucose	146.26 ^a^	83.02 ^b^	70.17 ^b^	165.44 ^a^
	Fructose	38.16 ^b^	32.01 ^c^	22.18 ^d^	74.61 ^a^
	Linoleic acid	31.46 ^b^	31.33 ^b^	33.29 ^b^	52.31 ^a^
	Linolenic acid	172.69 ^c^	295.52 ^b^	333.38 ^a^	60.84 ^d^
	Cafestol	108.16 ^c^	256.68 ^a^	146.62 ^b^	73.39 ^d^
	Kahweol	22.88 ^ab^	19.61 ^ab^	15.51 ^b^	32.27 ^a^
	Cinnamyl alcohol	10.40 ^bc^	7.77 ^c^	12.70 ^b^	19.28 ^a^
	Geraniol	530.22 ^b^	257.91 ^c^	175.31 ^d^	592.66 ^a^
	Pyrroline	5.49 ^b^	16.83 ^a^	1.81 ^b^	0.70 ^b^

H: High coffee cherry pulp-retention treatment; M: Medium coffee cherry pulp-retention treatment; L: Low coffee cherry pulp-retention treatment; N: Control treatment. All values are expressed as means (n = 3), and the unit is μg/g. Different lowercase letters indicate significant differences between treatments (*p* < 0.05).

**Table 2:** Entropy values, weights, and rankings of key flavor compounds in coffee.

	Indicator	Entropy Value (Hj)	Indicator Weight (ωj)	Rank
Bioactive	alkaloid	0.91	0.04	4
	organic acid	0.92	0.03	6
	flavonoids	0.98	0.01	26
	polyphenols	0.99	0.00	29
Flavor	pyridine	0.99	0.00	30
	pyrrole	0.87	0.05	3
	pyrazines	0.87	0.05	2
	alcohols	0.98	0.01	23
	phenols	0.97	0.01	18
	furan	0.99	0.01	27
	aldehydes	1.00	0.00	31
	acids	0.93	0.03	8
	indoles	0.98	0.01	28
	esters	0.92	0.03	7
Physical chemistry	hardness	0.98	0.01	25
	water content	0.99	0.00	32
	protein	0.97	0.01	21
	crude fat content	1.00	0.00	33
	sucrose	0.98	0.01	24
	soluble sugar	1.00	0.00	32
Sensory	Dry Aroma	0.94	0.03	9
	Wet Aroma	0.97	0.01	19
	Flavor	0.85	0.06	1
	Aftertaste	0.89	0.05	5
	Acidity	0.94	0.03	10
	Sweetness	0.93	0.03	11
	Mouthfeel	0.93	0.03	12
	Bitterness	0.94	0.02	13
	Body	0.93	0.03	14
	Cleanliness	0.96	0.02	20

**Table 3:** Results of the grey relational analysis of the quality of green coffee beans in different treatments.

Treatments	Equal-Weight Grey Relational Degree	Equal-Weight Grey Relational Degree Sort	Weighted Grey Relational Degree	Weighted Grey Relational Degree Sort
H	0.68	4	0.66	4
M	0.77	1	0.81	1
L	0.75	2	0.76	2
N	0.71	3	0.70	3

**Table 4:** Mean sensory evaluation scores and rankings of coffee samples under different treatment levels (H, M, L, N). The sensory profiles across ten attributes are illustrated in Figure 7.

Treatments	Dry Aroma	Wet Aroma	Flavor	Aftertaste	Acidity	Sweetness	Mouthfeel	Bitterness	Body	Cleanliness	Total Score	Rank
H	7.78	7.97	6.56	6.25	8.17	4.84	6.88	7.56	6.56	9.08	71.65	4
M	9.03	8.61	9.50	9.09	7.48	7.26	8.91	4.73	9.03	6.71	80.35	1
L	7.93	7.72	9.56	8.11	7.87	8.20	9.35	3.15	9.08	7.76	78.73	2
N	7.88	7.98	8.50	7.25	6.16	6.38	8.47	5.36	7.46	7.81	73.25	3

**Table 5:** Topological parameters of the correlation network among physicochemical properties, nutritional components, functional components, and volatile precursors in green coffee beans under different coffee cherry pulp-retention treatments (H, M, L, N).

Network Metrics	Relative Content of Physicochemical Properties, Different Functional and Volatile Flavor Precursors Components
Number of Nodes	20
Number of Edges	132
Number of Positive Correlations	64
Number of Negative Correlations	68
Percentage of Positive Edges	48.48%
P% from Nutritional Indicators to Functional Components	44.44%
P% from Nutritional Indicators to Volatile Components	57.89%
P% from Functional Components to Volatile Components	55.56%
P% between Nutritional Indicators	40.00%
P% between Functional Components	33.33%
P% between Volatile Components	38.89%
Average Connectivity (avgK)	3.96
Average Clustering Coefficient (avgCC)	0.33
Average Path Length (APL)	3.03
Graph Density	0.15

## Data Availability

The original data presented in the study are openly available in Mendeley Data, V1, doi: 10.17632/vcky9s3ktm.1.
